# Supplementary material for: Modeling glioblastoma heterogeneity as a dynamic network of cell states
Source: Mol Syst Biol. 2021 Sep 16;17(9):e10105. doi: 10.15252/msb.202010105 (PMC8444284; doi:10.15252/msb.202010105)
Supplement: Supplementary file 6 — Source Data for Figure 5 [file MSB-17-e10105-s004.zip › Figure5A_sourcedata/GSEA_3071/hallmarks_stateIV.GseaPreranked.1623742551702/HALLMARK_MYOGENESIS.html]

Details for gene set HALLMARK\_MYOGENESIS[GSEA]

|  || Dataset | state53071 |
| Phenotype | NoPhenotypeAvailable |
| Upregulated in class | na\_neg |
| GeneSet | HALLMARK\_MYOGENESIS |
| Enrichment Score (ES) | -0.40093374 |
| Normalized Enrichment Score (NES) | -1.9636306 |
| Nominal p-value | 0.0 |
| FDR q-value | 0.0017775621 |
| FWER p-Value | 0.022 |
Table: GSEA Results Summary

  

Fig 1: Enrichment plot: HALLMARK\_MYOGENESIS      
 Profile of the Running ES Score & Positions of GeneSet Members on the Rank Ordered List

  

| PROBE | GENE SYMBOL | GENE\_TITLE | RANK IN GENE LIST | RANK METRIC SCORE | RUNNING ES | CORE ENRICHMENT || 1 | CRYAB |  |  | 15 | 2.373 | 0.0278 | No |
| 2 | CLU |  |  | 27 | 2.064 | 0.0524 | No |
| 3 | IGFBP3 |  |  | 59 | 1.794 | 0.0696 | No |
| 4 | CKB |  |  | 72 | 1.711 | 0.0894 | No |
| 5 | NCAM1 |  |  | 129 | 1.456 | 0.0975 | No |
| 6 | APP |  |  | 136 | 1.426 | 0.1148 | No |
| 7 | ITGA7 |  |  | 148 | 1.380 | 0.1305 | No |
| 8 | CDH13 |  |  | 158 | 1.349 | 0.1462 | No |
| 9 | PKIA |  |  | 204 | 1.253 | 0.1538 | No |
| 10 | CHRNA1 |  |  | 224 | 1.199 | 0.1657 | No |
| 11 | ERBB3 |  |  | 243 | 1.164 | 0.1773 | No |
| 12 | WWTR1 |  |  | 277 | 1.081 | 0.1849 | No |
| 13 | EPHB3 |  |  | 483 | 0.828 | 0.1563 | No |
| 14 | MRAS |  |  | 491 | 0.823 | 0.1656 | No |
| 15 | MYL6B |  |  | 529 | 0.786 | 0.1687 | No |
| 16 | GABARAPL2 |  |  | 583 | 0.739 | 0.1680 | No |
| 17 | NOTCH1 |  |  | 666 | 0.678 | 0.1611 | No |
| 18 | EIF4A2 |  |  | 860 | 0.575 | 0.1315 | No |
| 19 | NAV2 |  |  | 865 | 0.574 | 0.1382 | No |
| 20 | STC2 |  |  | 972 | 0.535 | 0.1248 | No |
| 21 | KCNH2 |  |  | 1000 | 0.522 | 0.1264 | No |
| 22 | SORBS1 |  |  | 1099 | 0.492 | 0.1139 | No |
| 23 | PRNP |  |  | 1110 | 0.488 | 0.1183 | No |
| 24 | EFS |  |  | 1205 | 0.463 | 0.1063 | No |
| 25 | CHRNB1 |  |  | 1217 | 0.459 | 0.1101 | No |
| 26 | SLC6A8 |  |  | 1365 | 0.424 | 0.0874 | No |
| 27 | HSPB8 |  |  | 1372 | 0.423 | 0.0917 | No |
| 28 | MAPRE3 |  |  | 1397 | 0.417 | 0.0925 | No |
| 29 | ATP6AP1 |  |  | 1419 | 0.412 | 0.0938 | No |
| 30 | PTP4A3 |  |  | 1480 | 0.395 | 0.0874 | No |
| 31 | MEF2A |  |  | 1547 | 0.379 | 0.0797 | No |
| 32 | SYNGR2 |  |  | 1577 | 0.371 | 0.0789 | No |
| 33 | HSPB2 |  |  | 1616 | 0.365 | 0.0763 | No |
| 34 | AKT2 |  |  | 1663 | 0.356 | 0.0721 | No |
| 35 | AGL |  |  | 1699 | 0.349 | 0.0699 | No |
| 36 | TSC2 |  |  | 2042 | 0.290 | 0.0081 | No |
| 37 | FKBP1B |  |  | 2122 | 0.281 | -0.0034 | No |
| 38 | CACNA1H |  |  | 2448 | -0.263 | -0.0623 | No |
| 39 | MYLK |  |  | 2532 | -0.273 | -0.0747 | No |
| 40 | FGF2 |  |  | 2613 | -0.284 | -0.0863 | No |
| 41 | ITGB1 |  |  | 2817 | -0.308 | -0.1213 | No |
| 42 | FLII |  |  | 2959 | -0.327 | -0.1441 | No |
| 43 | SVIL |  |  | 3218 | -0.363 | -0.1888 | No |
| 44 | LAMA2 |  |  | 3235 | -0.366 | -0.1872 | No |
| 45 | IFRD1 |  |  | 3599 | -0.429 | -0.2512 | No |
| 46 | KIFC3 |  |  | 3684 | -0.441 | -0.2616 | No |
| 47 | MEF2C |  |  | 3894 | -0.485 | -0.2954 | No |
| 48 | HBEGF |  |  | 3905 | -0.488 | -0.2910 | No |
| 49 | KLF5 |  |  | 4165 | -0.559 | -0.3335 | No |
| 50 | SOD3 |  |  | 4227 | -0.574 | -0.3377 | No |
| 51 | IGFBP7 |  |  | 4331 | -0.607 | -0.3496 | No |
| 52 | TPM3 |  |  | 4442 | -0.651 | -0.3623 | No |
| 53 | MYO1C |  |  | 4578 | -0.725 | -0.3788 | No |
| 54 | SMTN |  |  | 4603 | -0.734 | -0.3739 | No |
| 55 | SH3BGR |  |  | 4691 | -0.790 | -0.3804 | No |
| 56 | PDE4DIP |  |  | 4715 | -0.804 | -0.3744 | No |
| 57 | PDLIM7 |  |  | 4768 | -0.838 | -0.3735 | No |
| 58 | TGFB1 |  |  | 4781 | -0.846 | -0.3649 | No |
| 59 | TEAD4 |  |  | 4954 | -1.024 | -0.3846 | No |
| 60 | SPHK1 |  |  | 5040 | -1.169 | -0.3858 | Yes |
| 61 | LSP1 |  |  | 5095 | -1.278 | -0.3797 | Yes |
| 62 | GADD45B |  |  | 5099 | -1.292 | -0.3635 | Yes |
| 63 | MYH9 |  |  | 5132 | -1.366 | -0.3520 | Yes |
| 64 | NQO1 |  |  | 5178 | -1.556 | -0.3405 | Yes |
| 65 | COL15A1 |  |  | 5208 | -1.691 | -0.3242 | Yes |
| 66 | COL4A2 |  |  | 5235 | -1.917 | -0.3045 | Yes |
| 67 | TPM2 |  |  | 5242 | -1.971 | -0.2801 | Yes |
| 68 | COL6A2 |  |  | 5258 | -2.256 | -0.2538 | Yes |
| 69 | BIN1 |  |  | 5267 | -2.432 | -0.2239 | Yes |
| 70 | SSPN |  |  | 5273 | -2.514 | -0.1924 | Yes |
| 71 | TAGLN |  |  | 5285 | -3.021 | -0.1555 | Yes |
| 72 | COL3A1 |  |  | 5287 | -3.508 | -0.1103 | Yes |
| 73 | COL6A3 |  |  | 5288 | -3.654 | -0.0631 | Yes |
| 74 | COL1A1 |  |  | 5290 | -4.897 | -0.0000 | Yes |
Table: GSEA details [plain text format]

  

Fig 2: HALLMARK\_MYOGENESIS: Random ES distribution      
 Gene set null distribution of ES for **HALLMARK\_MYOGENESIS**
